# Supplementary material for: Intracellular artificial supramolecules based on de novo designed Y15 peptides
Source: Nat Commun. 2021 Jun 7;12:3412. doi: 10.1038/s41467-021-23794-6 (PMC8185068; doi:10.1038/s41467-021-23794-6)
Supplement: Supplementary file 1 — Supplementary Information [file 41467_2021_23794_MOESM1_ESM.pdf]

Supplementary Information for

Intracellular artificial supramolecules based on de novo designed Y15 peptides

Takayuki Miki*, Taichi Nakai, Masahiro Hashimoto, Keigo Kajiwara, Hiroshi Tsutsumi & Hisakazu Mihara

School of Life Science and Technology, Tokyo Institute of Technology, 4259 Nagatsuta-cho, Midori-ku, Yokohama, Kanagawa 226-8501, Japan

Table of Contents

Supplementary Tables 1-3 lists of oligo DNAs S3
Supplementary Table 4 Sequence of proteins used for in vitro experiments S4
Supplementary Table 5 Sequence of proteins used for mammalian cell expressions S5
Supplementary Figure 1 MALDI-TOF mass spectra of synthesized peptides S10
Supplementary Figure 2. Thioflavin T fluorescence intensity of Yn peptides at various concentrations S11
Supplementary Figure 3. Structure of Yn peptide assemblies S12
Supplementary Figure 4. Properties of Y15 self-assembling..S13
Supplementary Figure 5. Effect of ionic strength on Y15 assembling S14
Supplementary Figure 6 Assembling propensity of purified Yn-sfGFPs in test tubes S15
Supplementary Figure 7 DLS measurements of Yn-sfGFP derivatives S16
Supplementary Figure 8 Negative-stained TEM images of Y15-sfGFP S17
Supplementary Figure 9 Fluorescence anisotropy measurements of Yn-sGFP derivatives S18
Supplementary Figure 10 Colocalization of Y15-sfGFP and Y15-mCherry-HA in living HEK293 cells S19

Supplementary Figure 11 Properties of Y15-sfGFP self-assembly in cells S20
Supplementary Figure 12 Impact of Y15-tagged site on self-assembly S21
Supplementary Figure 13 Sedimentation assay of Y15-tagged fluorescent proteins in cell lysates ...S22
Supplementary Figure 14 Correlative light and electron microscopy of Y15-AG expressing COS-7 cells S23
Supplementary Figure 15 Properties of Y15-AG-HA assemblies in cells S24
Supplementary Figure 16 Effects of Y15-AG-HA on cell toxicity S25
Supplementary Figure 17 Fluorescence observation of HEK293 cells expressing Y15-mCherry-HA S26
Supplementary Figure 18 Immunofluorescence images of COS-7 cells expressing Y15-AG-HA and Y15-mCherry-Nck S27
Supplementary Figure 19 CLSM observation of COS-7 cells expressing Y15-AG-HA and mCherry-Nck S28
Supplementary Figure 20 CLSM images of polymerized actin and co-assemblies of Y15-AG-HA and Y15-mCherry-Nck series in COS-7 cells S29
Supplementary Figure 21 Correlation between dose of plasmids and composition of cytosolic granules S30
Supplementary Figure 22 Density dependency of Y15-mCherry-Nck on actin polymerization S31

Supplementary Tables

Supplementary Table 1. oligo DNAs for construction of Yn-sfGFP

Peptide	Fragment	Forward Sequence	Reverse Sequence
Y9	F1	ctagcgccaccatgtacgaatataaatacg	tattcgtatttatattcgtacatggtggcg
	F2	aatataaatacggcggtggcg	tcgacgccaccgccgtattta
Y11	F1	ctagcgccaccatgtacgaatataaatacg	tattcgtatttatattcgtacatggtggcg
	F2	aatataaatacgaatatggcggtggcg	tcgacgccaccgccatattcgtattta
Y13	F1	ctagcgccaccatgtacgaatataaatacg	tattcgtatttatattcgtacatggtggcg
	F2	aatataaatacgaatataaatacggcggtggc g	tcgacgccaccgccgtatttatattcgtattta
Y15	F1	ctagcgccaccatgtacgaatataaatacg	tattcgtatttatattcgtacatggtggcg
	F2	aatataaatacgaatataaatacgaatatggcg gtggcg	tcgacgccaccgccatattcgtatttatattcgta ttta
Y15(K9P)	F1	ctagcgccaccatgtacgaatataaatacg	tattcgtatttatattcgtacatggtggcg
	F2	aatatccgtacgaatataaatacgaatatggcg gtggcg	aattcgccaccgccatattcgtatttatattcgta cgga

Supplementary Table 2. oligo DNAs for construction of Yn-sfGFP-HA or Yn-sfGFP-CAAX

Peptide	Fragment	Forward Sequence	Reverse Sequence
Y15	F1	ctagcgccaccatgtacgaatataaatacg	tattcgtatttatattcgtacatggtggcg
	F2	aatataaatacgaatataaatacgaatatggcg gtggcg	aattcgccaccgccatattcgtatttatattcgtat tta
Y15(K9P)	F1	ctagcgccaccatgtacgaatataaatacg	tattcgtatttatattcgtacatggtggcg
	F2	aatatccgtacgaatataaatacgaatatggcg gtggcg	aattcgccaccgccatattcgtatttatattcgta cgga
HA		tcgactacccatacgacgtgccagactacgct ggttatccgtatgatgtacctgattatgcatgag c	ggccgctcatgcataatcaggtacatcatacgg ataaccagcgtagtctggcacgtcgtatgggta g
NES		tcgacctgcctcccctggagcgcctgaccctg gactaagc	ggccgcttagtccagggtcaggcgctccagg ggaggcagg

CAAX (K-RAS)	tcgacaagaagaagaagaagagcaagacca agtgcgtgatcatgtaagc	ggccgcttacatgatcacgcacttggtcttgctc ttcttcttcttcttg

Supplementary Table 3. primers for cDNA cloning of Nck1

Protein	Fragment	Forward Sequence	Reverse Sequence
Nck1 full	1st PCR	cgggaagtgctgaagctgctgaaag	cacagcagtcacttctggtcagcag
	2nd PCR	gaatctagaatggcagaagaagtggtggt	cttgcggccgctcatgataaatgcttgaca
Nck (1-258)		gaagtcgacatggcagaagaagtggtgg	ctgcggccgctcaacctgaagttaatggat
Nck (109-258)		gttgtcgacatgcccgcttatgtgaaatt	ctgcggccgctcaacctgaagttaatggat

Supplementary Table 4. Sequence of proteins used for in vitro experiments

Protein	Sequence
sfGFP	MGSSHHHHHHSSGLVPRGSHMASATMGGGVDMVSKGEELFTGVVPIL VELDGDVNGHKFSVRGEGEGDATNGKLTLKFICTTGKLPVPWPTLVTT LTYGVQCFSRYPDHMKQHDFFKSAMPEGYVQERTISFKDDGTYKTRAE VKFEGDTLVNRIELKGIDFKEDGNILGHKLEYNFNSHNVYITADKQKNG IKANFKIRHNVEDGSVQLADHYQQNTPIGDGPVLLPDNHYLSTQSVLSK DPNEKRDHMVLLEFVTAAGITHGMDELYK
Y9-sfGFP	MGSSHHHHHHSSGLVPRGSHMASATMYEYKYEYKYGGGVDMVSKGE ELFTGVVPILVELDGDVNGHKFSVRGEGEGDATNGKLTLKFICTTGKLP VPWPTLVTTLTYGVQCFSRYPDHMKQHDFFKSAMPEGYVQERTISFKD DGTYKTRAEVKFEGDTLVNRIELKGIDFKEDGNILGHKLEYNFNSHNV YITADKQKNGIKANFKIRHNVEDGSVQLADHYQQNTPIGDGPVLLPDN HYLSTQSVLSKDPNEKRDHMVLLEFVTAAGITHGMDELYK
Y11-sfGFP	MGSSHHHHHHSSGLVPRGSHMASATMYEYKYEYKYEYGGGVDMVSK GEELFTGVVPILVELDGDVNGHKFSVRGEGEGDATNGKLTLKFICTTGK LPVPWPTLVTTLTYGVQCFSRYPDHMKQHDFFKSAMPEGYVQERTISF KDDGTYKTRAEVKFEGDTLVNRIELKGIDFKEDGNILGHKLEYNFNSH

	NVYITADKQKNGIKANFKIRHNVEDGSVQLADHYQQNTPIGDGPVLLP DNHYLSTQSVLSKDPNEKRDHMVLLEFVTAAGITHGMDELYK
Y13-sfGFP	MGSSHHHHHHSSGLVPRGSHMASATMYEYKYEYKYEYKYGGGVDMV SKGEELFTGVVPILVELDGDVNGHKFSVRGEGEGDATNGKLTLKFICTT GKLPVPWPTLVTTLTYGVQCFSRYPDHMKQHDFFKSAMPEGYVQERTI SFKDDGTYKTRAEVKFEGDTLVNRIELKGIDFKEDGNILGHKLEYNFNS HNVYITADKQKNGIKANFKIRHNVEDGSVQLADHYQQNTPIGDGPVLL PDNHYLSTQSVLSKDPNEKRDHMVLLEFVTAAGITHGMDELYK
Y15-sfGFP	MGSSHHHHHHSSGLVPRGSHMASATMYEYKYEYKYEYKYEYGGGVD MVSKGEELFTGVVPILVELDGDVNGHKFSVRGEGEGDATNGKLTLKFIC TTGKLPVPWPTLVTTLTYGVQCFSRYPDHMKQHDFFKSAMPEGYVQER TISFKDDGTYKTRAEVKFEGDTLVNRIELKGIDFKEDGNILGHKLEYNF NSHNVYITADKQKNGIKANFKIRHNVEDGSVQLADHYQQNTPIGDGPV LLPDNHYLSTQSVLSKDPNEKRDHMVLLEFVTAAGITHGMDELYK
Y15(K9P)-sfGFP	MGSSHHHHHHSSGLVPRGSHMASATMYEYKYEYPYEYKYEYGGGVD MVSKGEELFTGVVPILVELDGDVNGHKFSVRGEGEGDATNGKLTLKFIC TTGKLPVPWPTLVTTLTYGVQCFSRYPDHMKQHDFFKSAMPEGYVQER TISFKDDGTYKTRAEVKFEGDTLVNRIELKGIDFKEDGNILGHKLEYNF NSHNVYITADKQKNGIKANFKIRHNVEDGSVQLADHYQQNTPIGDGPV LLPDNHYLSTQSVLSKDPNEKRDHMVLLEFVTAAGITHGMDELYK

Supplementary Table 5. Sequence of proteins used for mammalian cell expressions

Protein	Sequence
sfGFP	MVSKGEELFTGVVPILVELDGDVNGHKFSVRGEGEGDATNGKLTLKFIC TTGKLPVPWPTLVTTLTYGVQCFSRYPDHMKQHDFFKSAMPEGYVQER TISFKDDGTYKTRAEVKFEGDTLVNRIELKGIDFKEDGNILGHKLEYNF NSHNVYITADKQKNGIKANFKIRHNVEDGSVQLADHYQQNTPIGDGPV LLPDNHYLSTQSVLSKDPNEKRDHMVLLEFVTAAGITHGMDELYK
Y9-sfGFP	MYEYKYEYKYGGGVDMVSKGEELFTGVVPILVELDGDVNGHKFSVRG EGEGDATNGKLTLKFICTTGKLPVPWPTLVTTLTYGVQCFSRYPDHMKQ HDFFKSAMPEGYVQERTISFKDDGTYKTRAEVKFEGDTLVNRIELKGID

	FKEDGNILGHKLEYNFNSHNVYITADKQKNGIKANFKIRHNVEDGSVQ LADHYQQNTPIGDGPVLLPDNHYLSTQSVLSKDPNEKRDHMVLLEFVT AAGITHGMDELYK
Y11-sfGFP	MYEYKYEYKYEYGGGVDMVSKGEELFTGVVPILVELDGDVNGHKFSV RGEGEGDATNGKLTLKFICTTGKLPVPWPTLVTTLTYGVQCFSRYPDHM KQHDFFKSAMPEGYVQERTISFKDDGTYKTRAEVKFEGDTLVNRIELK GIDFKEDGNILGHKLEYNFNSHNVYITADKQKNGIKANFKIRHNVEDGS VQLADHYQQNTPIGDGPVLLPDNHYLSTQSVLSKDPNEKRDHMVLLEF VTAAGITHGMDELYK
Y13-sfGFP	MYEYKYEYKYEYKYGGGVDMVSKGEELFTGVVPILVELDGDVNGHK FSVRGEGEGDATNGKLTLKFICTTGKLPVPWPTLVTTLTYGVQCFSRYP DHMKQHDFFKSAMPEGYVQERTISFKDDGTYKTRAEVKFEGDTLVNRI ELKGIDFKEDGNILGHKLEYNFNSHNVYITADKQKNGIKANFKIRHNVE DGSVQLADHYQQNTPIGDGPVLLPDNHYLSTQSVLSKDPNEKRDHMV LLEFVTAAGITHGMDELYK
Y15-sfGFP	MYEYKYEYKYEYKYEYGGGVDMVSKGEELFTGVVPILVELDGDVNG HKFSVRGEGEGDATNGKLTLKFICTTGKLPVPWPTLVTTLTYGVQCFSR YPDHMKQHDFFKSAMPEGYVQERTISFKDDGTYKTRAEVKFEGDTLV NRIELKGIDFKEDGNILGHKLEYNFNSHNVYITADKQKNGIKANFKIRH NVEDGSVQLADHYQQNTPIGDGPVLLPDNHYLSTQSVLSKDPNEKRDH MVLLEFVTAAGITHGMDELYK
Y15(K9P)-sfGFP	MYEYKYEYPYEYKYEYGGGVDMVSKGEELFTGVVPILVELDGDVNGH KFSVRGEGEGDATNGKLTLKFICTTGKLPVPWPTLVTTLTYGVQCFSRY PDHMKQHDFFKSAMPEGYVQERTISFKDDGTYKTRAEVKFEGDTLVN RIELKGIDFKEDGNILGHKLEYNFNSHNVYITADKQKNGIKANFKIRHN VEDGSVQLADHYQQNTPIGDGPVLLPDNHYLSTQSVLSKDPNEKRDH MVLLEFVTAAGITHGMDELYK
Y15-mCherrty-HA	MYEYKYEYKYEYKYEYGGGEFMVSKGEEDNMAIIKEFMRFKVHMEG SVNGHEFEIEGEGEGRPYEGTQTAKLKVTKGGPLPFAWDILSPQFMYGS KAYVKHPADIPDYLKLSFPEGFKWERVMNFEDGGVVTVTQDSSLQDGE FIYKVKLRGTNFPSDGPVMQKKTMGWEASSERMYPEDGALKGEIKQR LKLKDGGHYDAEVKTTYKAKKPVQLPGAYNVNIKLDITSHNEDYTIVE QYERAEGRHSTGGMDELYKVDYPYDVPDYAGYPYDVPDYA

Y15-sfGFP-NES	MYEYKYEYKYEYKYEYGGGEFMVSKGEELFTGVVPILVELDGDVNGH KFSVRGEGEGDATNGKLTLKFICTTGKLPVPWPTLVTTLTYGVQCFSRY PDHMKQHDFFKSAMPEGYVQERTISFKDDGTYKTRAEVKFEGDTLVN RIELKGIDFKEDGNILGHKLEYNFNSHNVYITADKQKNGIKANFKIRHN VEDGSVQLADHYQQNTPIGDGPVLLPDNHYLSTQSVLSKDPNEKRDH MVLLEFVTAAGITHGMDELYKVDLPPLERLTLD
Y15-sfGFP-CAAX	MYEYKYEYKYEYKYEYGGGEFMVSKGEELFTGVVPILVELDGDVNGH KFSVRGEGEGDATNGKLTLKFICTTGKLPVPWPTLVTTLTYGVQCFSRY PDHMKQHDFFKSAMPEGYVQERTISFKDDGTYKTRAEVKFEGDTLVN RIELKGIDFKEDGNILGHKLEYNFNSHNVYITADKQKNGIKANFKIRHN VEDGSVQLADHYQQNTPIGDGPVLLPDNHYLSTQSVLSKDPNEKRDH MVLLEFVTAAGITHGMDELYKVDKKKKKSKTKCVIM
Y15-sfGFP-HA	MYEYKYEYKYEYKYEYGGGEFMVSKGEELFTGVVPILVELDGDVNGH KFSVRGEGEGDATNGKLTLKFICTTGKLPVPWPTLVTTLTYGVQCFSRY PDHMKQHDFFKSAMPEGYVQERTISFKDDGTYKTRAEVKFEGDTLVN RIELKGIDFKEDGNILGHKLEYNFNSHNVYITADKQKNGIKANFKIRHN VEDGSVQLADHYQQNTPIGDGPVLLPDNHYLSTQSVLSKDPNEKRDH MVLLEFVTAAGITHGMDELYKVDYPYDVPDYAGYPYDVPDYA
sfGFP-Y15-HA	MVSKGEELFTGVVPILVELDGDVNGHKFSVRGEGEGDATNGKLTLKFIC TTGKLPVPWPTLVTTLTYGVQCFSRYPDHMKQHDFFKSAMPEGYVQER TISFKDDGTYKTRAEVKFEGDTLVNRIELKGIDFKEDGNILGHKLEYNF NSHNVYITADKQKNGIKANFKIRHNVEDGSVQLADHYQQNTPIGDGPV LLPDNHYLSTQSVLSKDPNEKRDHMVLLEFVTAAGITHGMDELYKEFG GSGGYEYKYEYKYEYKYEYGGSVDYPYDVPDYAGYPYDVPDYA
sfGFP-Y15	MVSKGEELFTGVVPILVELDGDVNGHKFSVRGEGEGDATNGKLTLKFIC TTGKLPVPWPTLVTTLTYGVQCFSRYPDHMKQHDFFKSAMPEGYVQER TISFKDDGTYKTRAEVKFEGDTLVNRIELKGIDFKEDGNILGHKLEYNF NSHNVYITADKQKNGIKANFKIRHNVEDGSVQLADHYQQNTPIGDGPV LLPDNHYLSTQSVLSKDPNEKRDHMVLLEFVTAAGITHGMDELYKVDG GGYEYKYEYKYEYKYEY
Y15-mAG-HA	MYEYKYEYKYEYKYEYGGGEFMVSVIKPEMKIKLCMRGTVNGHNFVI EGEGKGNPYEGTQILDLNVTEGAPLPFAYDILTTVFQYGNRAFTKYPADI QDYFKQTFPEGYHWERSMTYEDQGICTATSNISMRGDCFFYDIRFDGTN

	FPPNGPVMQKKTLKWEPSTEKMYVEDGVLKGDVNMRLLLEGGGHYR<br>CDFKTTYKAKKEVRLPDAHKIDHRIEILKHDKDYNKVKLYENAVARYS<br>MLPSQAKVDYPYDVPDYAGYPYDVPDYA
Y15-AG-HA	MYEYKYEYKYEYKYEYGGGEFMVSVIKPEMKIKLCMRGTVNGHNFVI<br>EGEGKGNPYEGTQILDLNVTEGAPLPFAYDILTTVFQYGNRAFTKYPADI<br>QDYFKQTFPEGYHWERSMTYEDQGICTATSNISMRGDCFFYDIRFDGV<br>NFPPNGPVMQKKTLKWEPSTEKMYVRDGVLKGDVNMALLLEGGGHY<br>RCDFKTTYKAKKDVRLPDYHFVDHRIEILKHDKDYNKVKLYENAVARY<br>SMLPSQAKVDYPYDVPDYAGYPYDVPDYA
Y15-mCherry-<br>Nck(109-258)	MYEYKYEYKYEYKYEYGGGEFMVSKGEEDNMAIIKEFMRFKVHMEG<br>SVNGHEFEIEGEGEGRPYEGTQTAKLKVTKGGPLPFAWDILSPQFMYGS<br>KAYVKHPADIPDYLKLSFPEGFKWERVMNFEDGGVVTVTQDSSLQDGE<br>FIYKVKLRGTNFPSDGPVMQKKTMGWEASSERMYPEDGALKGEIKQR<br>LKLKDGGHYDAEVKTTYKAKKPVQLPGAYNVNIKLDITSHNEDYTIVE<br>QYERAEGRHSTGGMDELYKVDMPAYVKFNYMAEREDELSLIKGTKVI<br>VMEKCSDGWWRGSYNGQVGWFPSNYVTEEGDSPLGDHVGSLSEKLA<br>AVVNNLNTGQVLHVVQALYPFSSSNDEELNFEKGDVMDVIEKPENDPE<br>WWKCRKINGMVGLVPKNYVTVMQNNPLTSG
Y15-mCherry-<br>Nck(1-258)	MYEYKYEYKYEYKYEYGGGEFMVSKGEEDNMAIIKEFMRFKVHMEG<br>SVNGHEFEIEGEGEGRPYEGTQTAKLKVTKGGPLPFAWDILSPQFMYGS<br>KAYVKHPADIPDYLKLSFPEGFKWERVMNFEDGGVVTVTQDSSLQDGE<br>FIYKVKLRGTNFPSDGPVMQKKTMGWEASSERMYPEDGALKGEIKQR<br>LKLKDGGHYDAEVKTTYKAKKPVQLPGAYNVNIKLDITSHNEDYTIVE<br>QYERAEGRHSTGGMDELYKVDMAEEVVVVAKFDYVAQQEQELDIKK<br>NERLWLLDDSKSWWRVRNSMNKTGFVPSNYVERKNSARKASIVKNLK<br>DTLGIGKVKRKPSVPDSASPADDSFVDPGERLYDLNMPAYVKFNYMAE<br>REDELSLIKGTKVIVMEKCSDGWWRGSYNGQVGWFPSNYVTEEGDSP<br>LGDHVGSLSEKLAAVVNNLNTGQVLHVVQALYPFSSSNDEELNFEKGD<br>VMDVIEKPENDPEWWKCRKINGMVGLVPKNYVTVMQNNPLTSG
Y15-mCherry-<br>2xNck(1-258)	MYEYKYEYKYEYKYEYGGGEFMVSKGEEDNMAIIKEFMRFKVHMEG<br>SVNGHEFEIEGEGEGRPYEGTQTAKLKVTKGGPLPFAWDILSPQFMYGS<br>KAYVKHPADIPDYLKLSFPEGFKWERVMNFEDGGVVTVTQDSSLQDGE<br>FIYKVKLRGTNFPSDGPVMQKKTMGWEASSERMYPEDGALKGEIKQR

	LKLKDGGHYDAEVKTTYKAKKPVQLPGAYNVNIKLDITSHNEDYTIVE<br>QYERAEGRHSTGGMDELYKVDMAEEVVVVAKFDYVAQQEQELDIKK<br>NERLWLLDDSKSWWRVRNSMNKTGFVPSNYVERKNSARKASIVKNLK<br>DTLGIGKVKRKPSVPDSASPADDSFVDPGERLYDLNMPAYVKFNYMAE<br>REDELSLIKGTKVIVMEKCSDGWWRGSYNGQVGWFPSNYVTEEGDSP<br>LGDHVGSLSEKLAAVVNNLNTGQVLHVVQALYPFSSSNDEELNFEKGD<br>VMDVIEKPENDPEWWKCRKINGMVGLVPKNYVTVMQNNPLTSGSRM<br>AEEVVVVAKFDYVAQQEQELDIKKNERLWLLDDSKSWWRVRNSMNK<br>TGFVPSNYVERKNSARKASIVKNLKDTLGIGKVKRKPSVPDSASPADDS<br>FVDPGERLYDLNMPAYVKFNYMAEREDELSLIKGTKVIVMEKCSDGW<br>WRGSYNGQVGWFPSNYVTEEGDSPLGDHVGSLSEKLAAVVNNLNTGQ<br>VLHVVQALYPFSSSNDEELNFEKGDVMDVIEKPENDPEWWKCRKING<br>MVGLVPKNYVTVMQNNPLTSG

Supplementary Figure

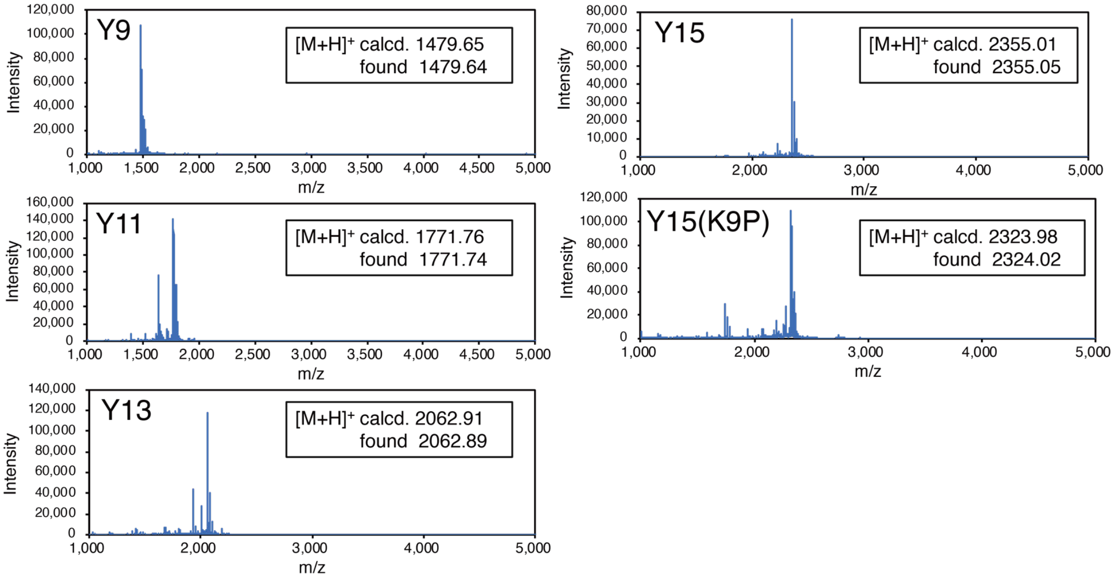

Supplementary Figure 1. MALDI-TOF mass spectra of synthesized peptides. Source data are provided as a Source Data file.

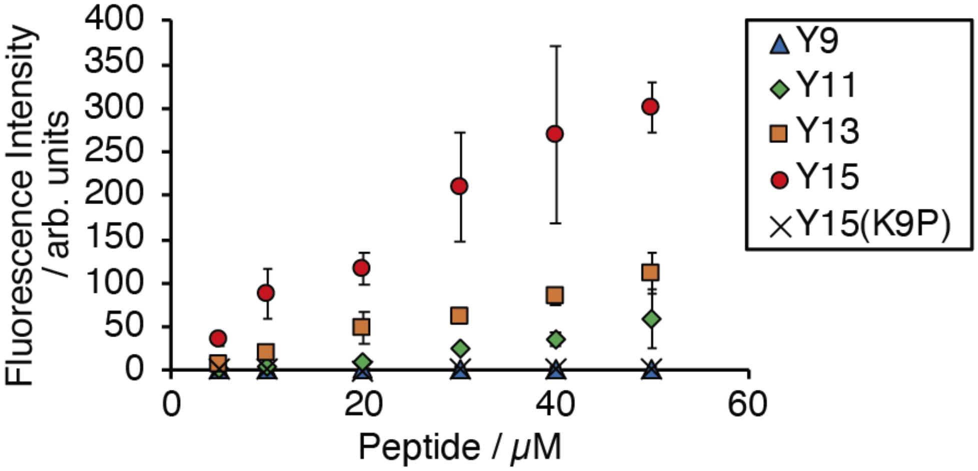

Supplementary Figure 2. Thioflavin T fluorescence intensity of Yn peptides at various concentrations. Peptides were incubated in PBS buffer for a day at 37 °C. To the peptide solutions (5–50 μM), thioflavin T (final 25 μM) was added. Data are presented as mean values +/- SD. (n = 3 biologically independent experiments). Source data are provided as a Source Data file.

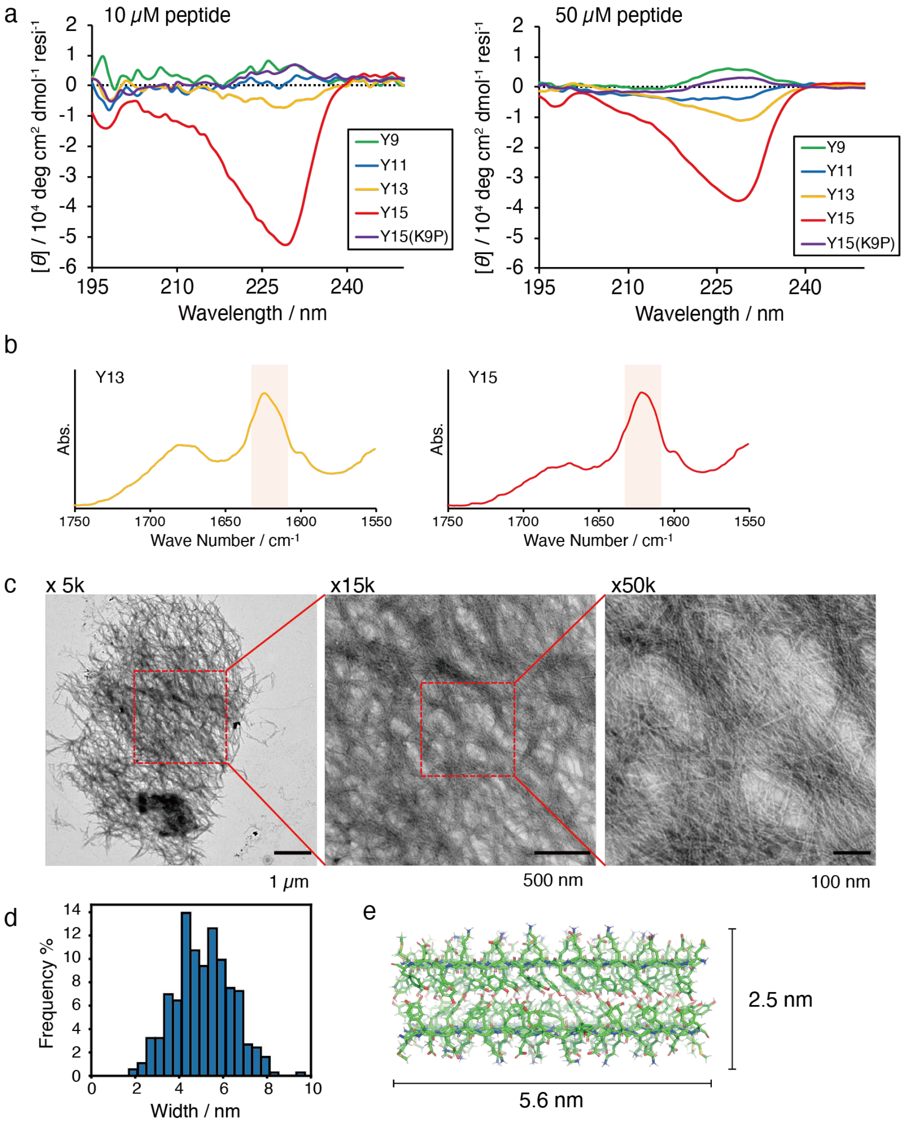

Supplementary Figure 3. Structure of Yn peptide assemblies. a CD spectra of Yn peptides. The peptides were dissolved in PBS buffer at 10 or 50 μM. The solutions were incubated for 1 day at 37 °C. **b** FT-IR spectra of Y13 or Y15 peptides. The peptide solutions (400 μM) in PBS buffer were dried, and these IR spectra were obtained. **c** Negative-stained TEM images of Y15 peptide. Y15 peptide (100 μM) in PBS was incubated for 22 hours at 37 °C. The mixture was attached to copper grids and negatively stained by nano-W. **d** Frequency of fibril width. Y15 formed nanofibers with a width of 5.0 ± 1.2 nm. **e** A plausible model of the Y15 peptide assembly. Here, we hypothesized that two antiparallel monolayers interact on Tyr hydrophobic faces. Source data are provided as a Source Data file.

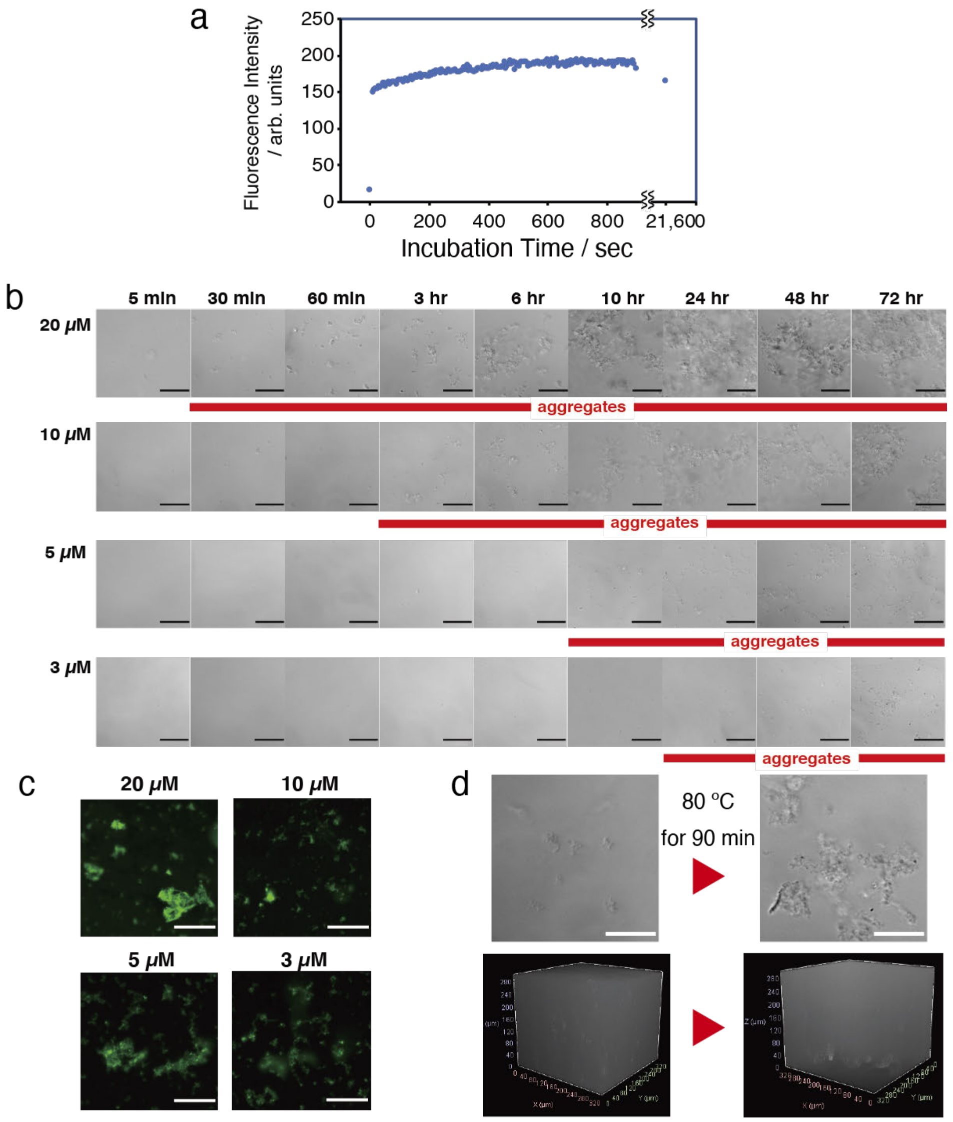

Supplementary Figure 4. Properties of Y15 self-assembling. a Time-course of thioflavin-T fluorescence intensity. To 25 μM of thioflavin-T solution in PBS, DMSO stock of Y15 peptide (4 mM) was added (final concentration is 20 μM). The fluorescence intensity was measured by F-7000 (Hitachi). Excitation, 430 nm (10 nm slit); Emission, 490 nm (20 nm slit). Source data are provided as a Source Data file. **b** DIC images of Y15 aggregation. Scale bar, 50 μm. **c** Thioflavin-T fluorescence images of Y15 aggregate. Thioflavin-T positive aggregates were clearly observed at all conditions (3, 5, 10, and 20 μM of Y15). Scale bar, 50 μm. **d** High temperature promoting Y15 aggregation. The 3D pictures show that Y15 precipitated at the bottom after heating (80 ºC for 90 min), whereas Y15 aggregates are floating before heating. Scale bar, 50 μm.

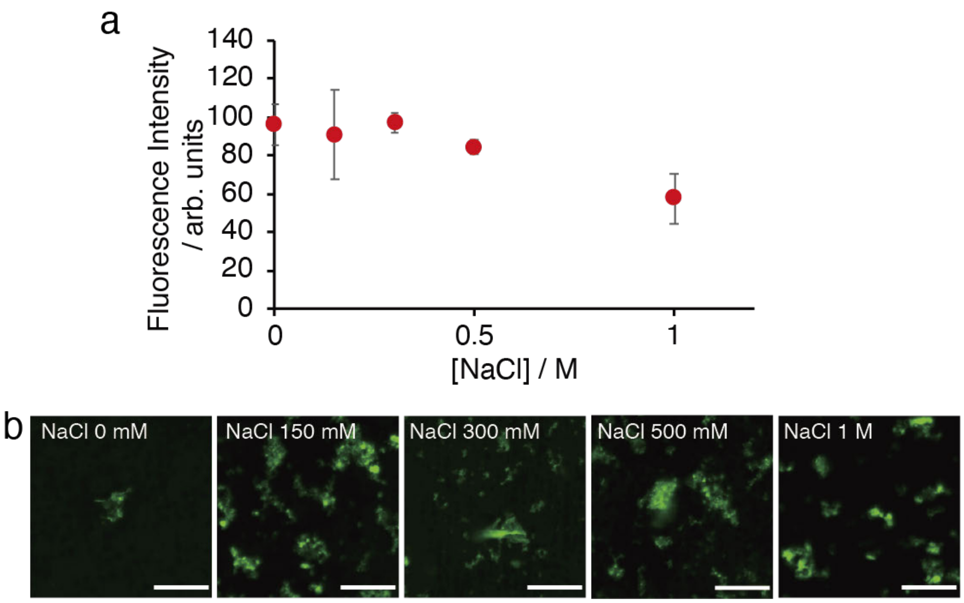

Supplementary Figure 5. Effect of ionic strength on Y15 assembling. a Thioflavin-T fluorescence intensity of Y15 assembly in different ion strength (*n* = 3 biologically independent experiments). To 25 μM of thioflavin-T solution in 10 mM sodium phosphate buffer (pH 7.2) with different concentration of NaCl (0, 150, 300, 500 and 1,000 mM), DMSO stock of Y15 peptide (2 mM) was added (final concentration is 10 μM). The fluorescence intensity was measured by F-7000 (Hitachi). Excitation, 430 nm (10 nm slit); Emission, 490 nm (20 nm slit). Data are presented as mean values +/- SD. Source data are provided as a Source Data file. **b** Thioflavin-T fluorescence images of Y15 aggregates. Thioflavin-T positive aggregates were clearly observed at all conditions (0, 150, 300, 500, and 1,000 mM NaCl). Scale bar, 50 μm.

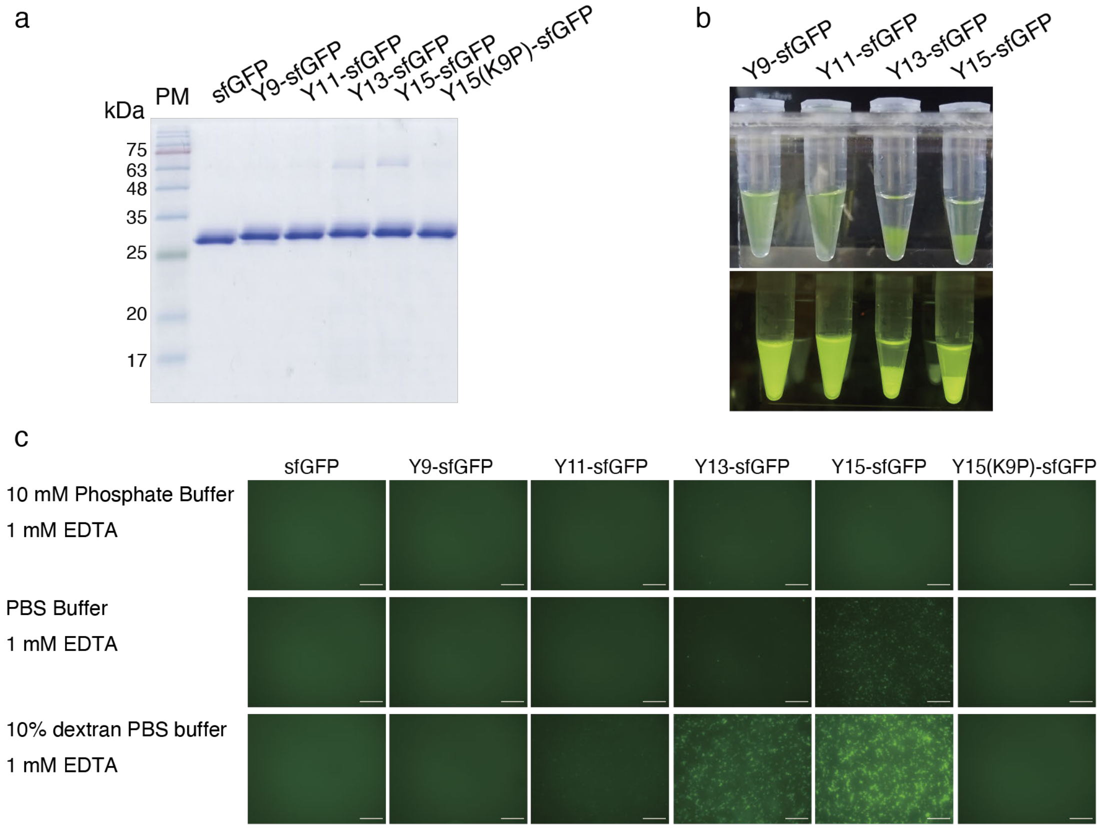

Supplementary Figure 6. Assembling propensity of purified Yn-sfGFPs in test tubes. a A CBB-stained gel image. The 5 μM of Yn-sfGFPs were mixed with 2×Laemmli buffer and loaded onto SDS-PAGE (12.5% acrylamide). The molecular weight of Yn-sfGFP was slightly increased due to tagging. In the case of Y13- or Y15-tagged sfGFP, dimer was slightly observed. Uncropped blots in Source Data. **b** Optical and fluorescence images of 15 μM of Yn-sfGFPs in 10 mM sodium phosphate buffer (pH 7.2, containing 1 mM EDTA). To obtain the fluorescence image, samples were excited by CyanoView (ATTO). Y13- and Y15-sfGFPs formed fluorescent protein condensates. **c** Fluorescence microscopic observation of 1 μM of Yn-sfGFP in different solutions (Scale bar, 100 μm). Self-assembling of Y15-sfGFP was promoted in the presence of a crowding agent (10w/v% of dextran 6,000).

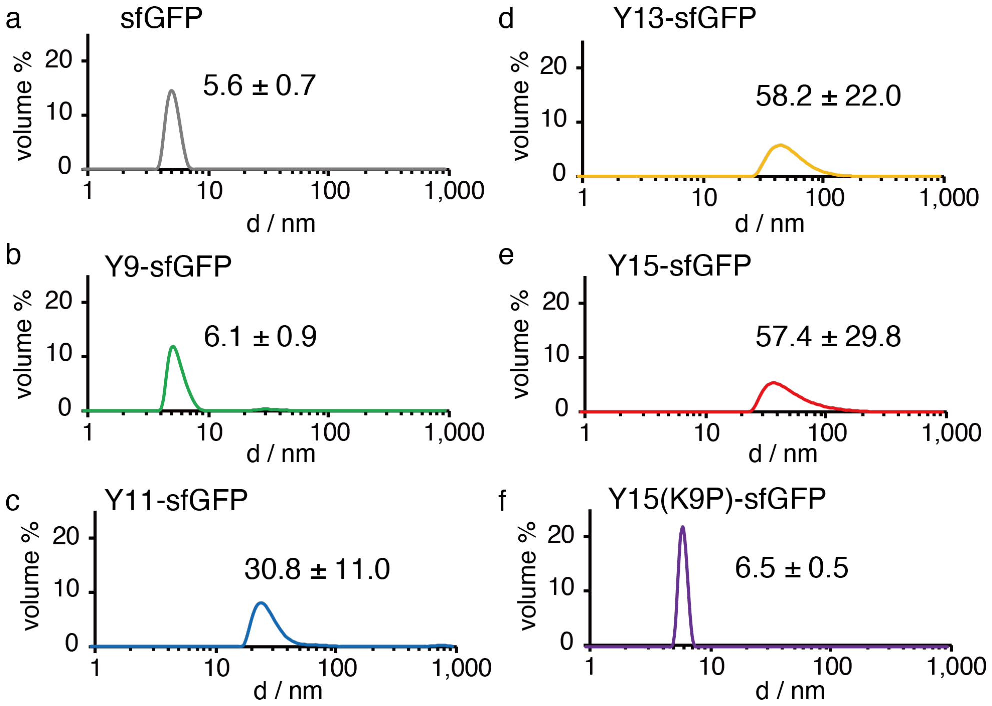

Supplementary Figure 7. DLS measurements of Yn-sfGFP derivatives. After dialysis, the protein solutions (5 μM) in 10 mM sodium phosphate buffer (pH 7.2, 1 mM EDTA) were incubated for 24 hours at 37 °C, then hydrodynamic diameters of Yn-sfGFPs were determined. (**a**) sfGFP, (**b**)Y9-sfGFP, (**c**)Y11-sfGFP, (**d**) Y13-sfGFP, (**e**) Y15-sfGFP, (**f**) Y15(K9P)-sfGFP. Source data are provided as a Source Data file.

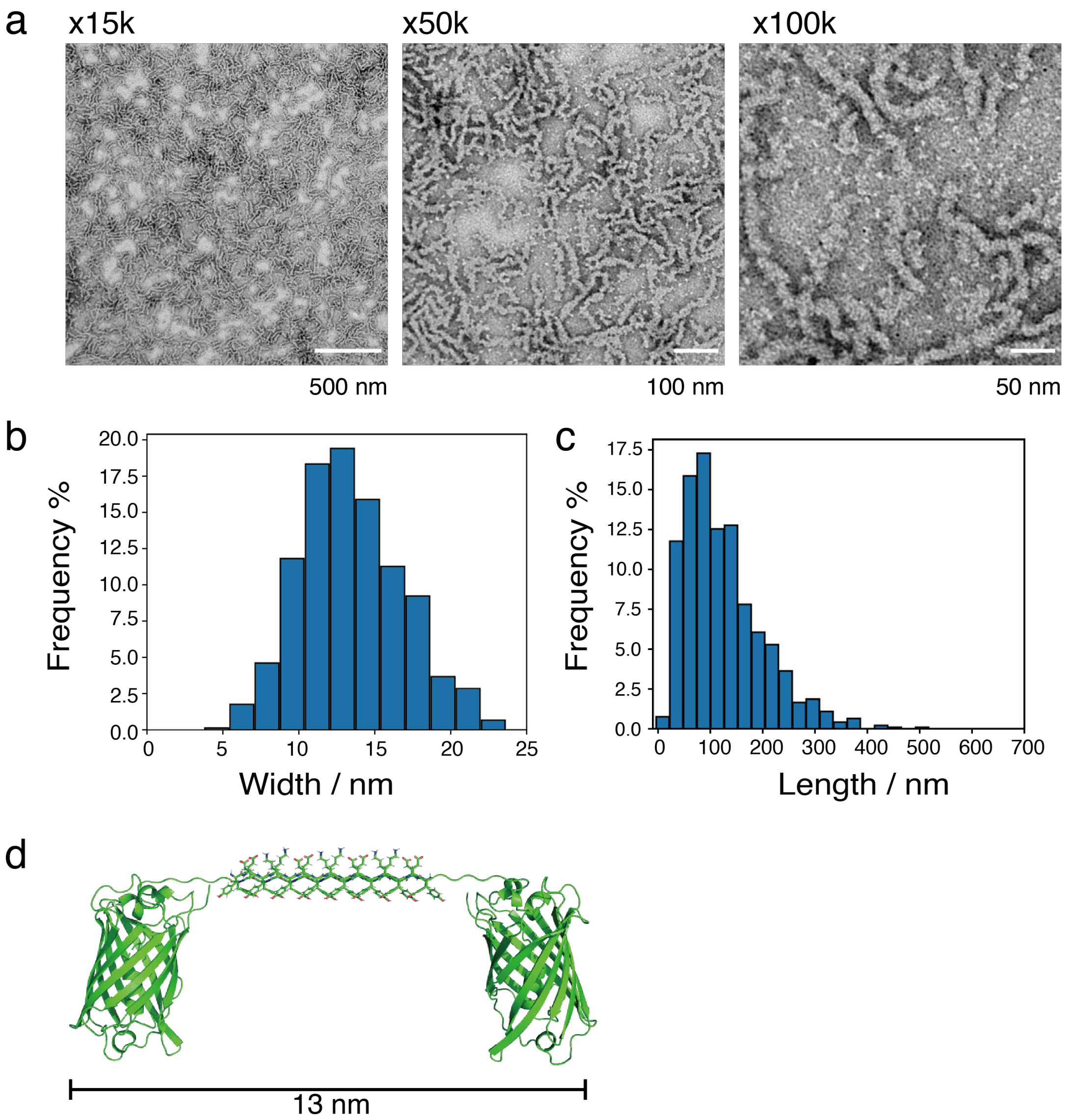

Supplementary Figure 8. Negative-stained TEM images of Y15-sfGFP. a Different magnitudes of TEM images of Y15-sfGFP. **b** Histogram distribution of fibril width. The mean diameter was 13.7 ± 3.2 nm. **c** Fibril length distribution. The mean length was 119 ± 71 nm. **d** Schematic illustration of the self-assembly of Y15-sfGFP. Here, we hypothesized an anti-parallel β-sheet structure of the Y15 tag. The distance between the edges is estimated to be approximately 13 nm. Source data are provided as a Source Data file.

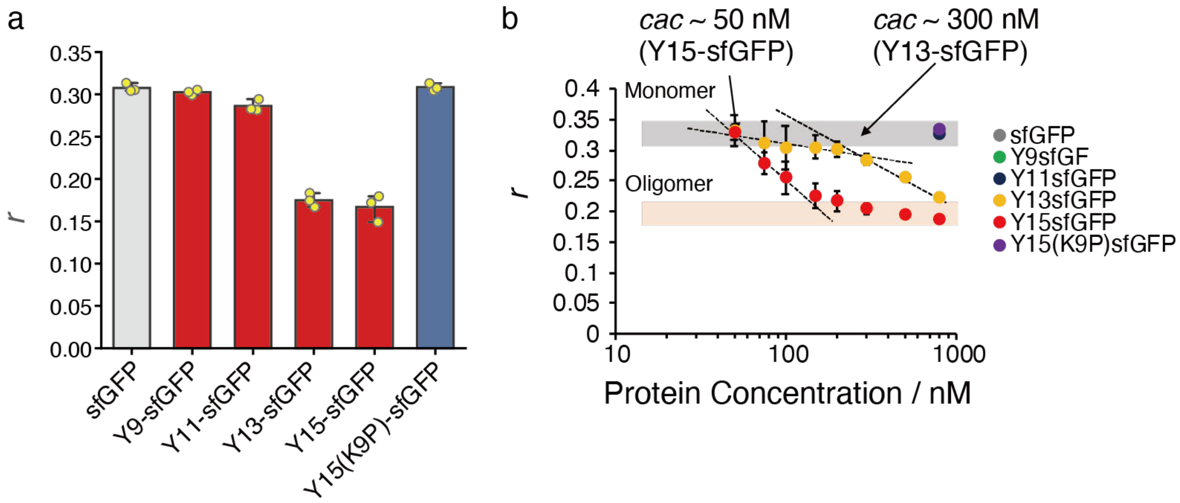

Supplementary Figure 9. Fluorescence anisotropy measurements of Yn-sGFP derivatives. a Y13- and Y15-sfGFP showed lower fluorescence anisotropy at 5 μM concentration (n = 3 biologically independent experiments). **b** Protein concentration dependency on fluorescence anisotropy (n = 3 biologically independent experiments). The denatured Yn-sfGFPs in 5 M urea buffer (10 mM sodium p hosphate, 1 mM EDTA) were 50-folded diluted with 10 mM sodium phosphate buffer with 1 mM EDTA and incubated for 1 day. The anisotropy of Y13-sfGFP dramatically decreased at 300 nM, whereas it atte nuated at 50 nM in the case of Y15-sfGFP. All data is presented as mean values +/- SD. Source data are provided as a Source Data file.

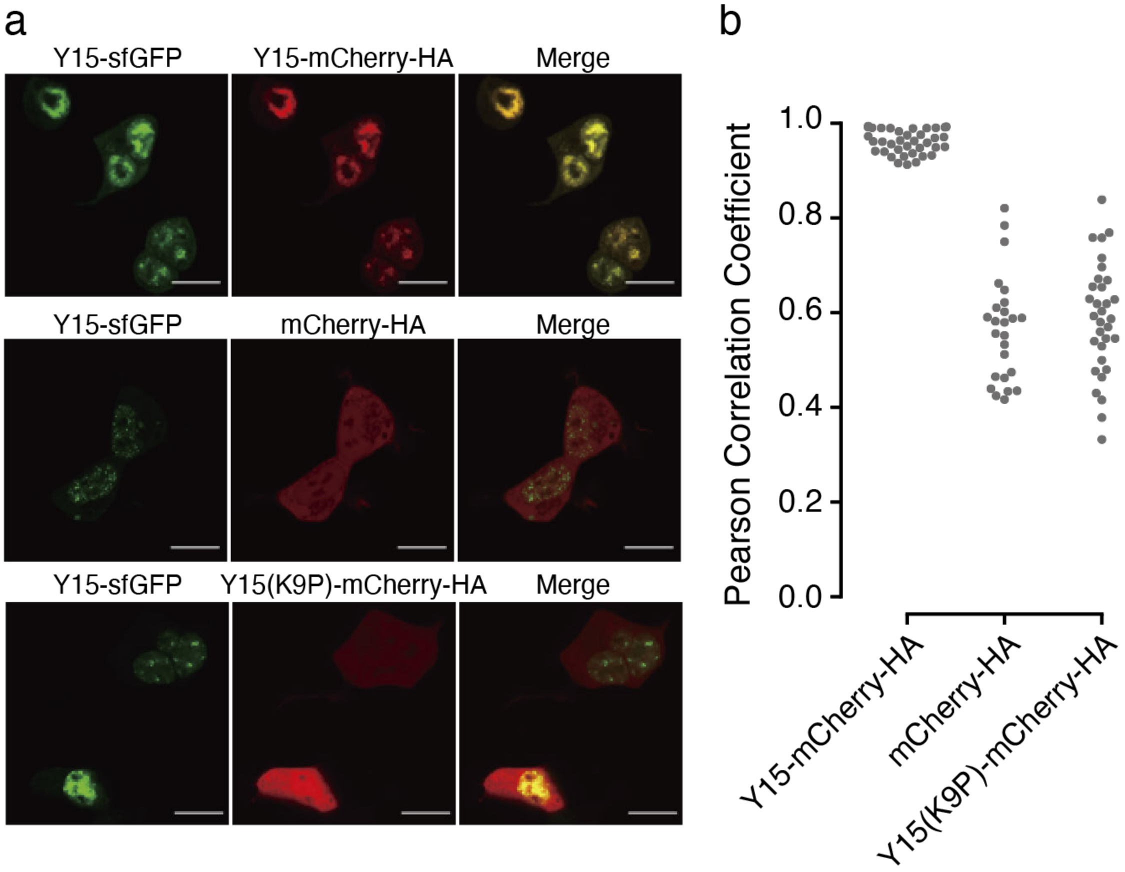

Supplementary Figure 10. Colocalization of Y15-sfGFP and Y15-mCherry-HA in living HEK293 cells. Cotransfection with a plasmid encoding Y15-sfGFP (250 ng) and a plasmid encoding Yn-mCherry-HA tag (250 ng) were performed. The cotransfected cells were observed by CLSM (**a**), and the Pearson correlation coefficient value for each cell was plotted (**b**). Scale bar, 10 μm. (n = 39, 25 and 32 cells examined over three biologically independent experiments for Y15-mCherry-HA, mCherry-HA, and Y15(K9P)-mCherry-HA, respectively). Source data are provided as a Source Data file.

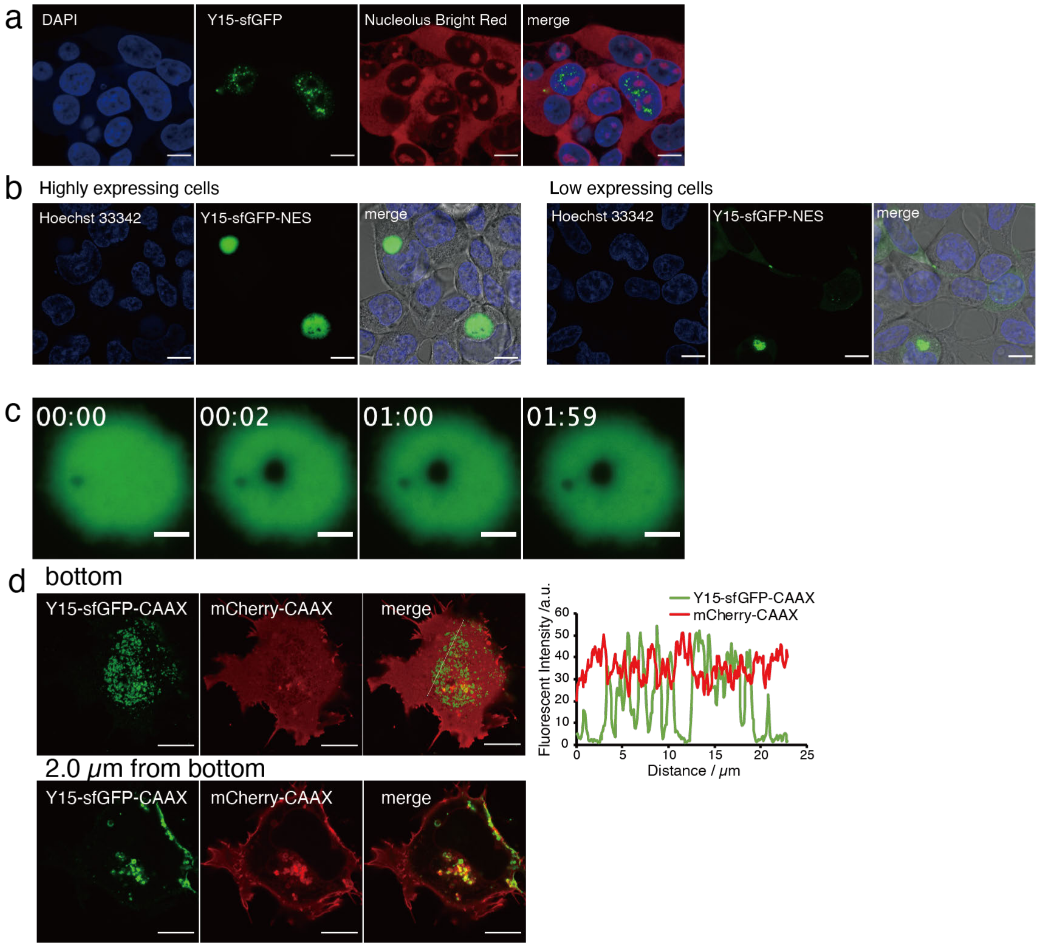

Supplementary Figure 11. Properties of Y15-sfGFP self-assembly in cells. a Nucleus localization of Y15-sfGFP. Transfected cells were fixed by 4% paraformaldehyde (PFA) phosphate buffer for 5 min and permeabilized with 0.5% Triton-X PBS for 20 min at room temperature, followed by washing with PBS. The cells were stained by DAPI and Nucleolus Bright Red reagent (MBL). Y15-sfGFP was assembled in the nucleus but not in the nucleolus. **b** Granular formation of Y15-sfGFP-NES in the cytoplasm. Transfected HEK293 cells were stained with Hoechst 33342 and observed by CLSM. Scale bar, 10 μm. **c** FRAP analysis of the Y15-sfGFP-NES granule in HEK293 cells. The fluorescence intensity in the bleached region was not recovered within 2 min. Scale bar, 2 μm. **d** CLSM observation of Y15-sfGFP-CAAX on HEK293 cells. Y15-sfGFP clusters on the inner leaflet were observed by CLSM (upper panel: bottom of the cell, lower panel: 2.0 μm height from bottom). In the right panel, the fluorescence intensity of the line is plotted, indicating that Y15-sfGFP-CAAX clusters excluded mCherry-CAAX to some extent. Scale bar, 10 μm. Source data are provided as a Source Data file.

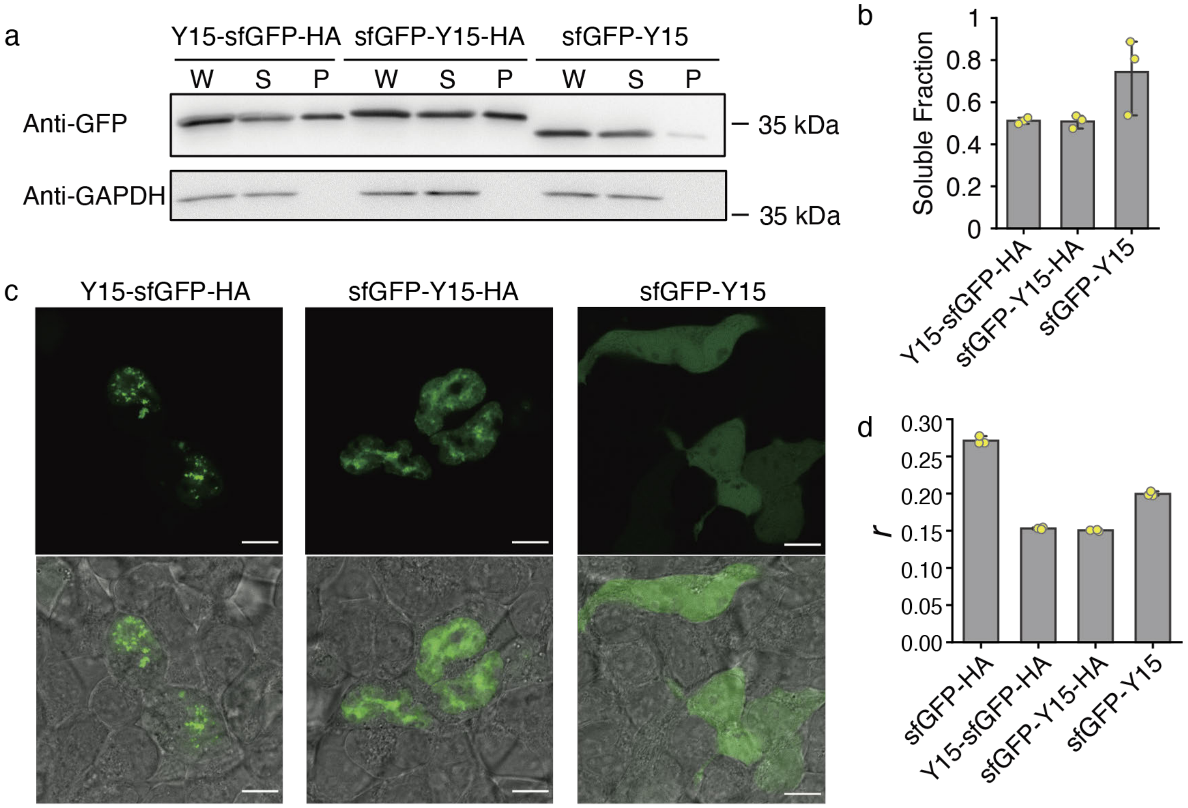

Supplementary Figure 12. Impact of Y15-tagged site on self-assembly. a-b, Sedimentation assay of cell lysate. The sfGFP derivatives were detected by an anti-sfGFP antibody for western blotting (**a**) (W; whole lysate, S; soluble fraction, P; pellet fraction). The ratio of the soluble fraction is shown in (**b**) (*n* = 3 biologically independent experiments). The sfGFP-Y15 terminating with the Y15 sequence was abunda

nt

in the soluble fraction compared to others. **c** CLSM images of transfected HEK293 cells. Y15-sfGFP-HA and sfGFP-Y15-HA protein concentrated as fluorescent granules, whereas sfGFP-Y15 was uniformly distributed in most cells. Scale bar, 10 μm. **d** Fluorescence anisotropy measurements of transfected living HEK293 cells (*n* = 3 biologically independent experiments). In fluorescence observation and anisotropy s Source data are provided as a Source Data file.ͻFP-Y15-HA. All data is presented as mean values +/- SD.

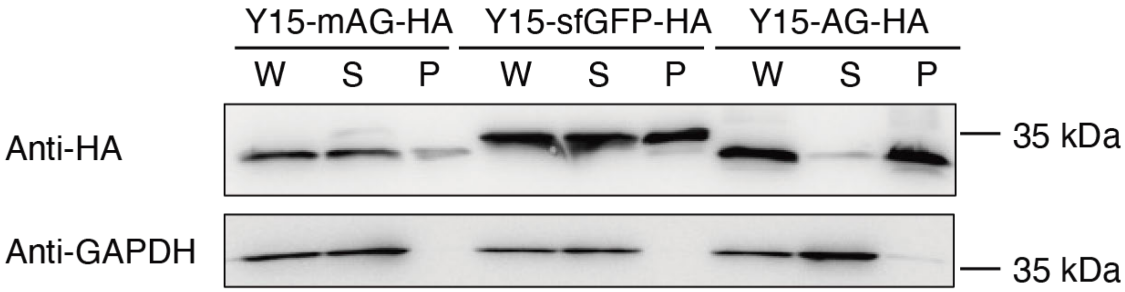

Supplementary Figure 13. Sedimentation assay of Y15-tagged fluorescent proteins in cell lysates. After lysis by Ripa buffer and centrifugation, samples were analyzed by western blotting using an anti-HA antibody. (W; whole lysate, S; soluble fraction, P; pellet fraction). Source data are provided as a Source Data file.

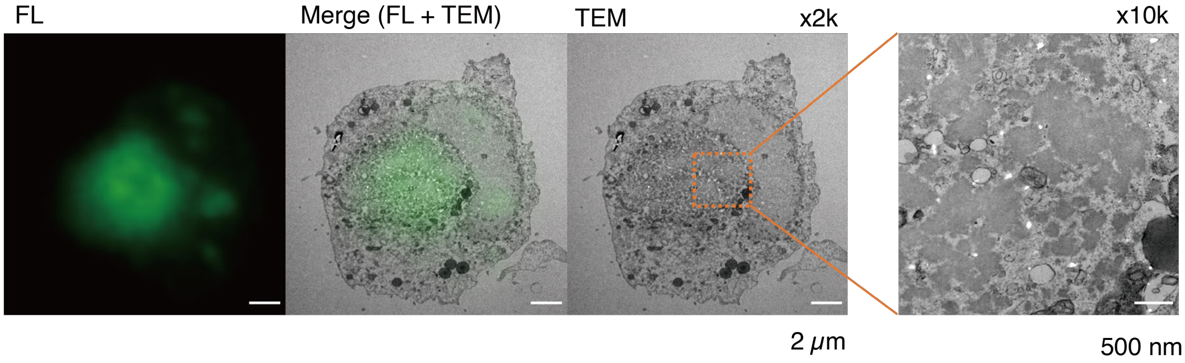

Supplementary Figure 14. Correlative light and electron microscopy of Y15-AG expressing COS-7 cells. Fluorescence image (left panel), overlay image (2nd panel), TEM image (3rd panel), and enlarged TEM image (right panel).

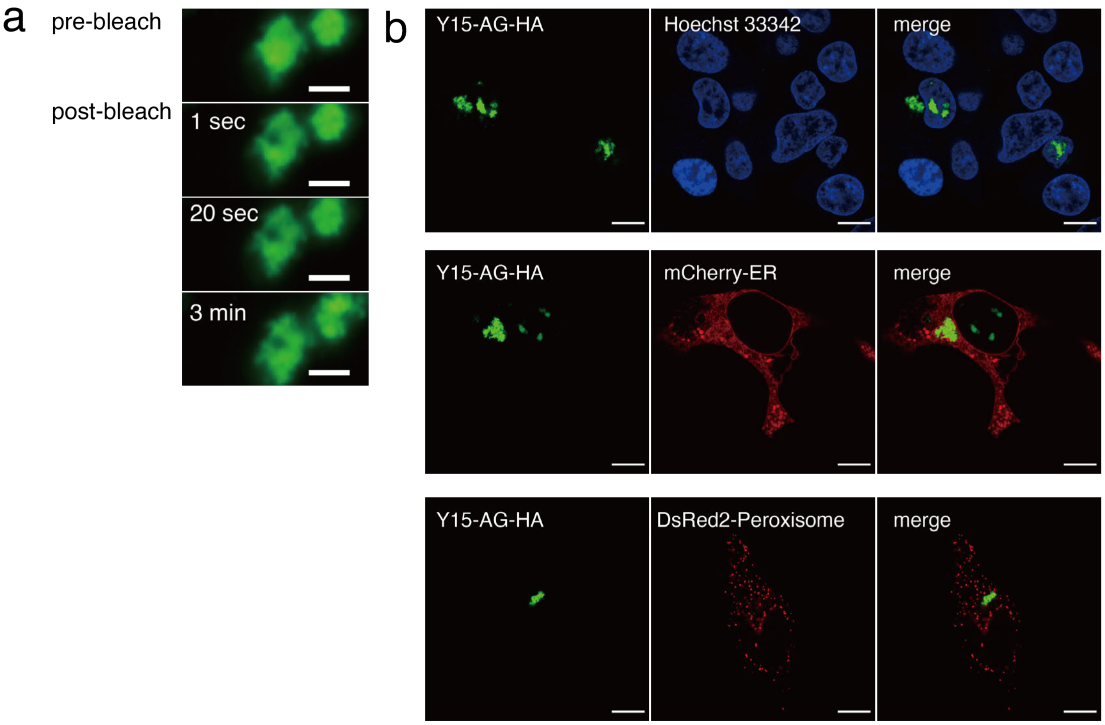

Supplementary Figure 15. Properties of Y15-AG-HA assemblies in cells. a FRAP analysis of Y15-AG clusters. Fluorescence in the bleached area was not recovered, suggesting that the assembly is rigid. Scale bar, 2 μm. **b** Subcellular localization of Y15-AG-HA clusters. Y15-AG-HA formed assemblies in the nucleus or adjacent to the ER. Scale bar, 10 μm.

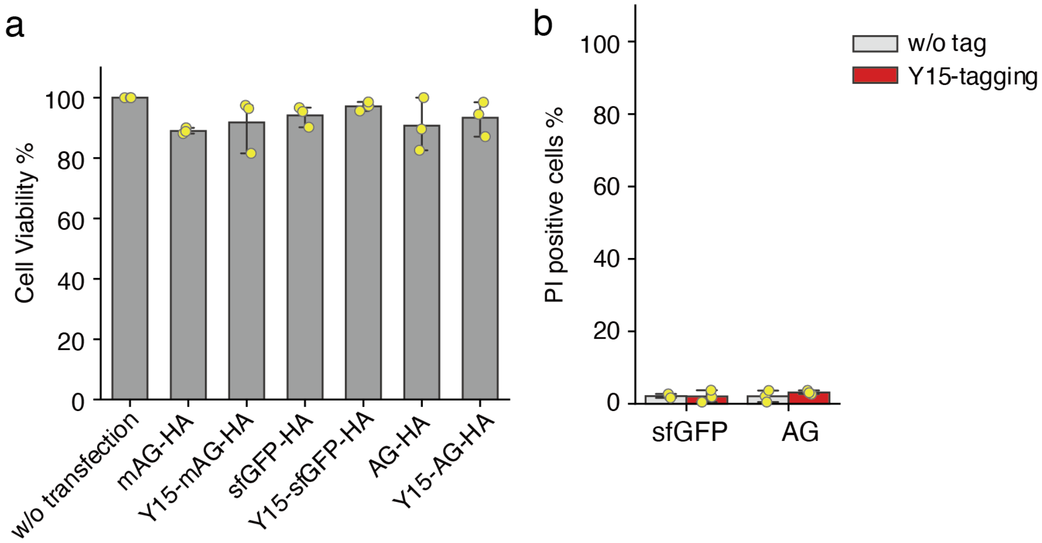

Supplementary Figure 16. Effects of Y15-AG-HA on cell toxicity. a Cell viability assay of transfected HEK293 cells (n = 3 biologically independent experiments). The viabilities were measured by Cel
l

Counting Kit-8 (Dojindo), 2 days after transfection. No significant difference between Y15-mAG-HA vs. mAG-HA (p = 0.64), Y15-sfGFP-HA vs. sfGFP-HA (p = 0.26), or Y15-AG-HA vs. AG-HA (p = 0.69) was observed in transfected HEK293 cells. **b** Low toxicity of Yn-protein expression. Dead cells of transfected HEK293 cells were stained by PI dye. The dead cells among transfected cells were counted (n = 3 biologically independent experiments). No significant difference between Y15-sfGFP-HA vs. sfGF P-HA (p = 0.94) or Y15-AG-HA vs. AG-HA (p = 0.37) was observed in transfected HEK293 cells. All d ata is presented as mean values +/- SD. p-values; two-tailed paired t-tests. Source data are provided as a S ource Data file.

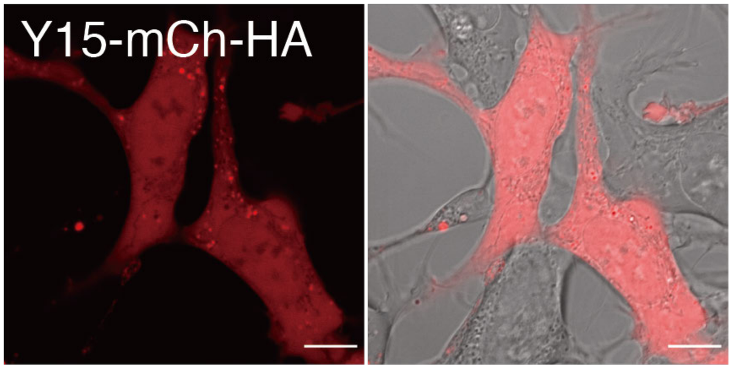

Supplementary Figure 17. Fluorescence observation of HEK293 cells expressing Y15-mCherry-HA. Scale bar, 10 μm.

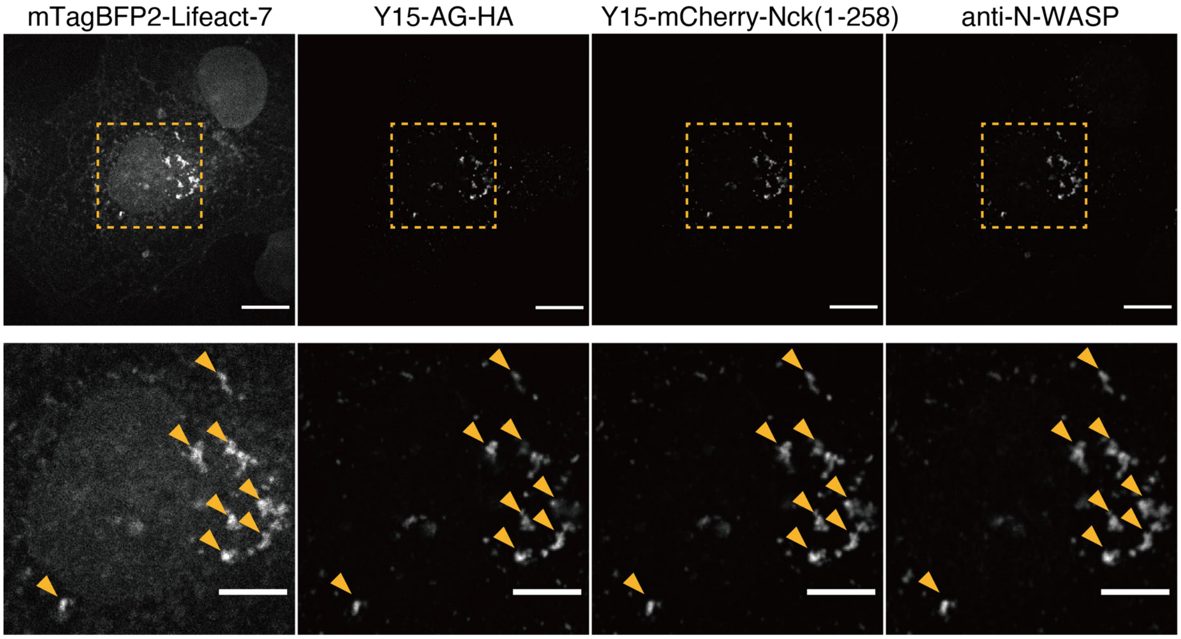

Supplementary Figure 18. Immunofluorescence images of COS-7 cells expressing Y15-AG-HA and Y15-mCherry-Nck. The lower panel showed the enlarged pictures of ROIs in the upper figure. Transfected cells on slide glass were fixed in 4% PFA, permeabilized by 0.5% Triton-X treatment, and incubated with 5% normal goat serum (NGS) PBS for 30 min at room temperature. After washing with PBS, the samples were incubated with a primary antibody (Novus, NBP1-82512) in 1% NGS PBS for overnight at 4 °C, washed with PBS, and incubated with a goat anti-rabbit IgG H&L (Alexa Fluor®647) (abcam, ab150079) in 1% NGS PBS for 1 hour at room temperature. Slides were mounted with ProLong Glass (Thermo). Scale bar, 10 μm (upper), 5 μm (lower).

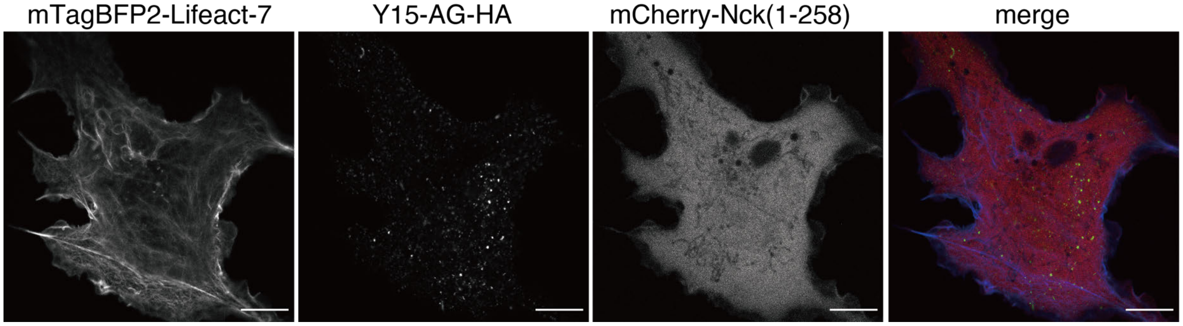

Supplementary Figure 19. CLSM observation of COS-7 cells expressing Y15-AG-HA and mCherry-Nck. The mCherry-Nck without Y15-tag (negative control) did not integrate in the Y15-AG-HA assembly. Scale bar, 10 μm.

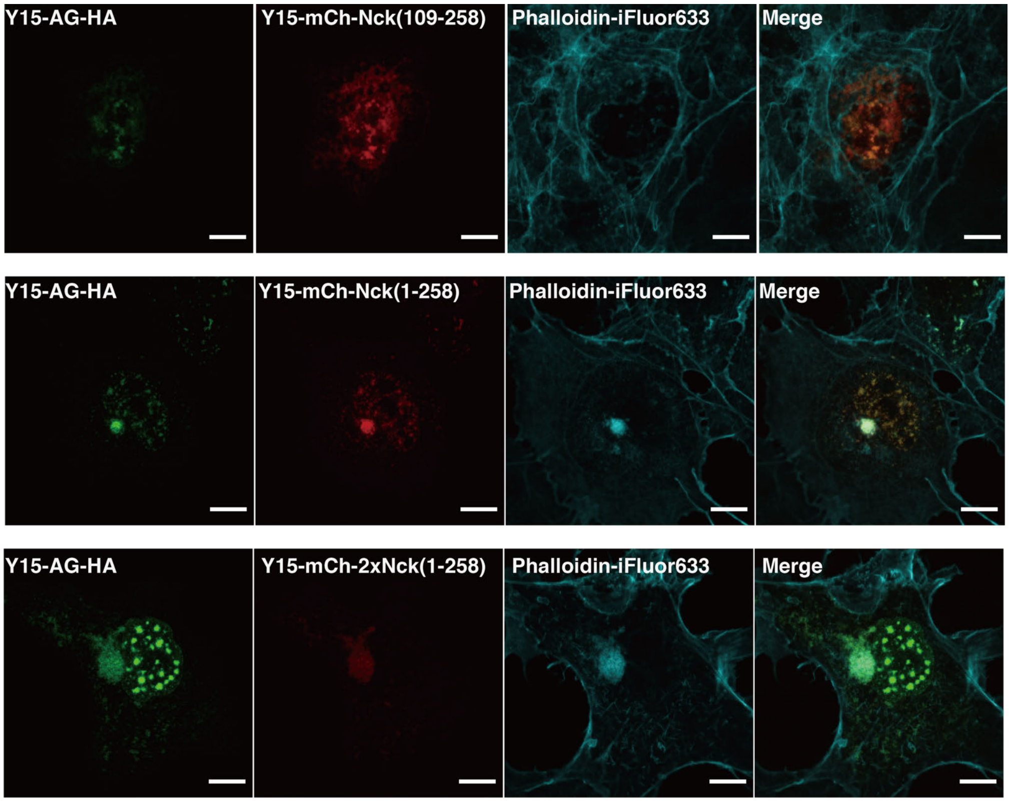

Supplementary Figure 20. CLSM images of polymerized actin and co-assemblies of Y15-AG-HA and Y15-mCherry-Nck series in COS-7 cells. The polymerized actin was stained by Phalloidin-iFluor 633 (abcam) after cell fixation and permeabilization. Scale bar, 10 μm.

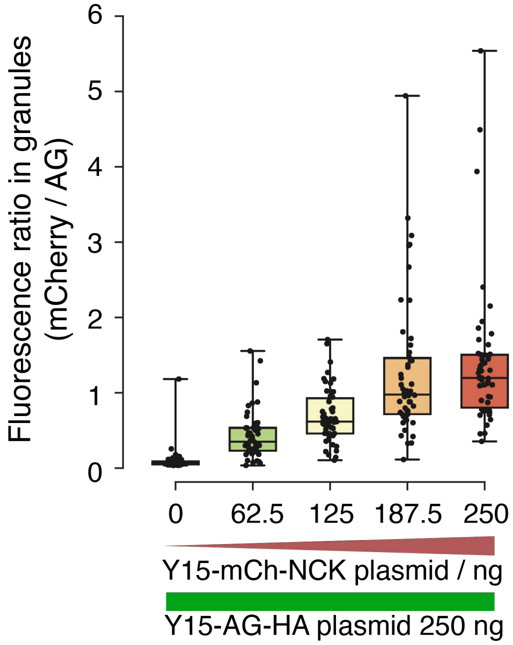

Supplementary Figure 21. Correlation between dose of plasmids and composition of cytosolic granules. The COS-7 cells transfected with constant amount of Y15-AG-HA plasmid (250 ng) and varying amount of Y15-mCherry-Nck plasmid (0-250 ng) were observed by CLSM. The ratio values were calculated using ImageJ. The boxplots are presented with the elements: center line, median; box limits, Q1 and Q3; whiskers, 1.5× interquartile range; points, outliers. ($n = 50$ cells examined over three biological independent experiments). Source data are provided as a Source Data file.

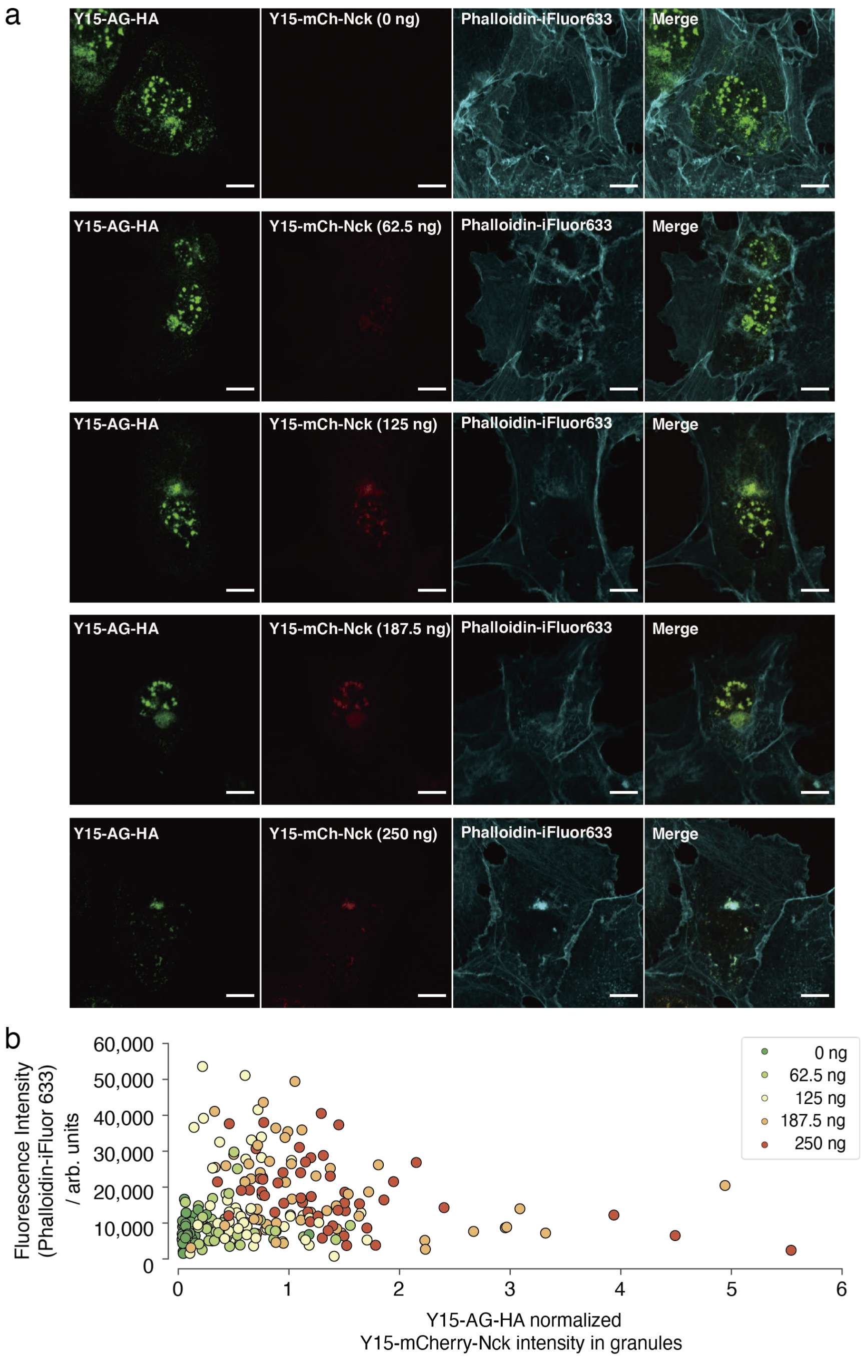

Supplementary Figure 22. Density dependency of Y15-mCherry-Nck on actin polymerization. a CLSM observation of the phalloidin-stained COS-7 cells with constant amount of Y15-AG-HA plasmid (250 ng) and increasing amount of Y15-mCherry-Nck plasmid (0-250 ng). Scale bar, 10 μm. **b** Relationship between actin intensities and Y15-mCherry-Nck density in granules. The individual plot

means the average fluorescence intensity of granules in single cell. The ratio values were calculated using ImageJ. (*n* = 50 cells examined over three biological independent experiments). Source data are provided as a Source Data file.
