# Supplementary material for: Intracellular artificial supramolecules based on de novo designed Y15 peptides
Source: Nat Commun. 2021 Jun 7;12:3412. doi: 10.1038/s41467-021-23794-6 (PMC8185068; doi:10.1038/s41467-021-23794-6)
Supplement: Supplementary file 3 — Reporting Summary [file 41467_2021_23794_MOESM3_ESM.pdf]

nature research

Corresponding author(s): Takayuki Miki

Last updated by author(s): May 7, 2021

Reporting Summary

Nature Research wishes to improve the reproducibility of the work that we publish. This form provides structure for consistency and transparency in reporting. For further information on Nature Research policies, see our Editorial Policies and the Editorial Policy Checklist.

Statistics

For all statistical analyses, confirm that the following items are present in the figure legend, table legend, main text, or Methods section.

n/a	Confirmed	
☐	☒	The exact sample size (*n*) for each experimental group/condition, given as a discrete number and unit of measurement
☐	☒	A statement on whether measurements were taken from distinct samples or whether the same sample was measured repeatedly
☐	☒	The statistical test(s) used AND whether they are one- or two-sided<br>*Only common tests should be described solely by name; describe more complex techniques in the Methods section.*
☒	☐	A description of all covariates tested
☒	☐	A description of any assumptions or corrections, such as tests of normality and adjustment for multiple comparisons
☐	☒	A full description of the statistical parameters including central tendency (e.g. means) or other basic estimates (e.g. regression coefficient) AND variation (e.g. standard deviation) or associated estimates of uncertainty (e.g. confidence intervals)
☐	☒	For null hypothesis testing, the test statistic (e.g. *F*, *t*, *r*) with confidence intervals, effect sizes, degrees of freedom and *P* value noted<br>*Give P values as exact values whenever suitable.*
☒	☐	For Bayesian analysis, information on the choice of priors and Markov chain Monte Carlo settings
☒	☐	For hierarchical and complex designs, identification of the appropriate level for tests and full reporting of outcomes
☒	☐	Estimates of effect sizes (e.g. Cohen's *d*, Pearson's *r*), indicating how they were calculated

Our web collection on statistics for biologists contains articles on many of the points above.

Software and code

Policy information about availability of computer code

Data collection	Fluorescence and DIC images,ZEN2.6 (blue) (ZEISS); CLSM images ZEN2.3 SP1 FP3 (Black) (ZEISS); TEM images,TEM CENTER (1.6.16.5152) (JEOL); CD measurements, spectra manager (2.8.0.4) (JASCO); Fluorescence anisotropy and fluorescence intensity, Wallac1420 Workstation (3.00) (PerkinElmer); Fluorescence spectra, FL Solutions (4.2) HITACHI; Gel images, ImageSaver6 (ATTO); DLS, Zetasizer Software (7.12) (Malvern)
Data analysis	Fiji/ImageJ (Version 2.1.0/1.53c) was used in all image data analysis. Plot figures were generated by Microsoft Excel (for Mac, Version 16.47.1) or Jupyter notebook (6.1.5) Python 3.7 with seaborn (0.11.0), pandas (1.1.5) and scipy(1.5.2) packages.

For manuscripts utilizing custom algorithms or software that are central to the research but not yet described in published literature, software must be made available to editors and reviewers. We strongly encourage code deposition in a community repository (e.g. GitHub). See the Nature Research guidelines for submitting code & software for further information.

Data

Policy information about availability of data

All manuscripts must include a data availability statement. This statement should provide the following information, where applicable:

- Accession codes, unique identifiers, or web links for publicly available datasets
- A list of figures that have associated raw data
- A description of any restrictions on data availability

The source data underlying all figures, including supplementary figures, are provided with this paper. The imaging data are available from the corresponding author upon reasonable request.

Field-specific reporting

Please select the one below that is the best fit for your research. If you are not sure, read the appropriate sections before making your selection.

- [x] Life sciences
- [ ] Behavioural & social sciences
- [ ] Ecological, evolutionary & environmental sciences

For a reference copy of the document with all sections, see nature.com/documents/nr-reporting-summary-flat.pdf

Life sciences study design

All studies must disclose on these points even when the disclosure is negative.

Sample size	The sample size for biochemical studies was chosen to be 3. For cell image analysis, we obtained 5 to 10 different images with different fields of view in a single biological experiment. We counted 25-50 cells for each conditions according to the appropriate literature (Salman F. B. et. al., Cell 166, 651-663 (2016)).
Data exclusions	No experimental data was excluded.
Replication	All experiments were replicated at least three times independently on different days. All attempts at replication were successful.
Randomization	In cell experiments, we used the same passages of cells to compare the protein differences. The independent experiments were performed at least three times on different days, and different passages of cells were used for replication. All obtained results were allocated for experimental group.
Blinding	The investigators were not blinded during fluorescence imaging experiments, because the transfection efficiency is not 100% . Only cells emitting fluorescence signals were allocated for experiments. All fluorescent cells observed were photographed one after another by changing fields of view, and all images were used for analysis. Imaging data were analyzed semi-automatically as written in the "methods" section.

Reporting for specific materials, systems and methods

We require information from authors about some types of materials, experimental systems and methods used in many studies. Here, indicate whether each material, system or method listed is relevant to your study. If you are not sure if a list item applies to your research, read the appropriate section before selecting a response.

Materials & experimental systems

n/a	Involved in the study	
[ ]	[x]	Antibodies
[ ]	[x]	Eukaryotic cell lines
[x]	[ ]	Palaeontology and archaeology
[x]	[ ]	Animals and other organisms
[x]	[ ]	Human research participants
[x]	[ ]	Clinical data
[x]	[ ]	Dual use research of concern

Methods

n/a	Involved in the study	
[x]	[ ]	ChIP-seq
[x]	[ ]	Flow cytometry
[x]	[ ]	MRI-based neuroimaging

Antibodies

Antibodies used	rabbit anti-GFP antibody (GeneTex, GTX113617); rabbit anti-HA-tag antibody (MBL, 561); mouse anti-GAPDH antibody (MBL, M171-3); goat anti-rabbit IgG-HRP conjugate (abcam, ab6721); goat anti-mouse IgG-HPR conjugate (Promega, W402B); mouse anti-HA antibody (MBL, M180-3S); rabbit anti-N-WASP antibody (Novus, NBP1-82512); goat anti-rabbit IgG H&L (Alexa Fluor®647) (abcam, ab150079)
Validation	All antibodies were commercially available and validated in manufacture's data sheets.<br>mouse anti-GAPDH antibody (MBL, M171-3); validated for human GAPDH in western blotting application (https://ruo.mbl.co.jp/bio/dtl/A/?pcd=M171-3MS)<br>rabbit anti-N-WASP antibody (Novus, NBP1-82512); validated for human and mouse N-WASP for immunofluorescence application (https://www.novusbio.com/products/n-wasp-antibody_nbp1-82512).

Eukaryotic cell lines

Policy information about cell lines

Cell line source(s)	HEK293 cells and COS-7 cells were provided by the RIKEN BRC through the National BioResource Project of the MEXT/AMED, Japan.

Authentication	All cells lines used in this study were not authentication.
Mycoplasma contamination	All cells lines used in this study were verified to be negative.
Commonly misidentified lines (See ICLAC register)	All cells lines used in this study were not misidentified lines.
